# Nasopharyngeal Carcinoma Presenting With Occipital Headache as a Sole Symptom in a Young Adult Male: A Case Report

**DOI:** 10.7759/cureus.37801

**Published:** 2023-04-18

**Authors:** Eid H Alshahrani, Abdulmalik Ismail

**Affiliations:** 1 Otolaryngology - Head and Neck Surgery, King Fahad University Hospital, Imam Abdulrahman Bin Faisal University, Khobar, SAU; 2 Otolaryngology - Head and Neck Surgery, Security Forces Hospital, Riyadh, SAU

**Keywords:** npc, fossa of rosenmuller, epstein-barr virus, headache, nasopharyngeal carcinoma

## Abstract

Nasopharyngeal carcinoma (NPC) is a rare type of cancer that is one of the most challenging cancers to diagnose correctly at the initial phase because of the different irrelative symptoms. Headache per se is rare and maybe a misleading symptom for diagnosing NPC. We report a case of a 37-year-old Saudi civil servant male with NPC who presented to the clinic complaining of a continuous, dull occipital headache that is progressively worsened over the last three months, not responding to over-the-counter analgesics. Computed tomography revealed a large ill-defined infiltrative heterogeneously enhancing soft tissue mass that obliterated the fossae of Rosenmuller and pharyngeal openings of both Eustachian tubes. The histopathological diagnosis was undifferentiated non-keratinizing NPC that is positive for Epstein-Barr virus. As in this case, headache alone can be the sole presenting symptom for NPC. Therefore, physicians should think broader in such a presentation to appropriately diagnose and treat NPC.

## Introduction

Nasopharyngeal carcinoma (NPC) is rare cancer affecting the nasopharynx. It is quite rare in most parts of the world, with an estimated incidence of 129,079 cases and 72,987 associated deaths in 2018. The majority are geographically localized to South East Asia and are likely to show an upward trend annually [1]. In terms of demographics, men are two to three times more likely to develop the disease than women, and the peak age of disease incidence is between 50 and 60 years, with a minor peak observed among adolescents and young adults in Southeast Asia, the Middle East/North Africa, and the United States [2,3]. A large body of evidence supports the role of the Epstein-Barr virus (EBV) as the primary etiologic agent in the pathogenesis of NPC, but the geographic variation suggests a multifactorial etiology. Other than EBV (the most common risk factor), human papillomavirus (HPV), smoking, alcohol uptake, preserved foods, and genetic predisposition have all been shown to have an apparent association with NPC occurrence [4-6]. According to the fifth edition of the World Health Organization (WHO) classification of head and neck tumors, NPC is classified into three categories: non-keratinizing squamous cell carcinoma (SCC), keratinizing SCC, and basaloid SCC [7]. Non-keratinizing tumors are further subcategorized as undifferentiated or differentiated [7].

The nonspecificity of NPC presenting symptoms and diagnostic difficulties is attributed to the late diagnosis and, thus, limited therapeutic options and late management of the disease [8,9]. The presenting symptoms include but are not limited to neck mass (75.8%; most common presentation), unilateral ear block or reduced hearing (62.4%), recurrent epistaxis (44.6%), infrequently, nonspecific headaches (34.8%), dysphagia (21.4%), tinnitus (23.2%), facial numbness (17.9%), diplopia (16.1%), nasal obstruction (10.7%), and weight loss (6.9%). The NPC diagnosis is often delayed because of the aforementioned reasons [8,10]. Around 96% of NPC patients present with multiple symptoms, while only 0.3% presented with headaches alone [10]. Evaluation of NPC should include history and physical examination that includes cranial nerves and nasopharyngoscopy. Imaging studies include computed tomography (CT) and magnetic resonance imaging (MRI) of the nasopharynx, skull base, and neck [2]. The definitive diagnosis is made by histopathological examination of the primary tumor [2]. Other useful investigations for confirming a diagnosis of NPC are quantitative assessments of plasma immune serology and EBV DNA [2]. At the time of diagnosis, the majority of patients had advanced disease (stages III and IV: 13.7% and 65.5%, respectively) [11-13]. Radiotherapy is the primary treatment modality for early stage disease, while radiotherapy with concurrent chemotherapy is deemed the mainstay treatment in advanced cases [4]. Variable regimens of platinum-based concurrent chemotherapy are used in clinical practice, but cisplatin is commonly the first choice [2,4,13,14]. Nasopharyngectomy is generally reserved for recurrent disease as a last resort, given the proximity of the nasopharynx to the brain stem, major blood vessels, and cranial nerves. Neck dissection is indicated for the residual nodal disease after initial radiotherapy or isolated neck recurrence [15]. At present, ongoing trials are investigating new advances such as molecular-targeted therapies, immunotherapy, and minimally invasive surgeries showing promising results [15,16].

We report here the case of a young adult male diagnosed with an undifferentiated non-keratinizing NPC, who initially presented solely with nonspecific, chronic headaches for many months. The patient tried self-medication with over-the-counter analgesics. Finally, when his symptoms became unbearable, he saw a neurologist, who obtained an MRI, which revealed an extensive nasopharyngeal mass extending the skull base and cavernous sinus and encasing the carotid artery. The biopsy confirmed the diagnosis of NPC, and the case was discussed in the tumor board for appropriate management. Informed consent was obtained from the patient to publish this case report.

## Case presentation

A 37-year-old Saudi civil servant male was referred to the Ear, Nose, and Throat (ENT) clinic from a neurology clinic in a private hospital after a suspicious brain MRI. The patient presented to the clinic complaining of a continuous, dull occipital headache that is progressively worsening over the last three months, not radiating elsewhere, and not responding to over-the-counter pain medications. He reported no fever, night sweating, loss of appetite, or loss of weight. He had no hearing loss, aural fullness, otalgia, nasal obstruction, epistaxis, or any other ENT or neurological symptoms. The patient had no family history of malignancies. He had a 20 pack years smoking history but had otherwise no known NPC risk factors.

Examination revealed a well-built and nourished young male. Ear examination was normal, with no effusion with a positive Rinne's test bilaterally, with no laterality on Weber's test. Throat and oral examinations were normal, with the tongue and palate movement intact. Flexible nasopharyngoscopy revealed an irregular mass in the nasopharynx obscuring the fossae Rosenmuller. He had palpable anterior cervical lymphadenopathy on neck examination. Examination of the cranial nerves from I to XII was unremarkable.

**Figure 1:**
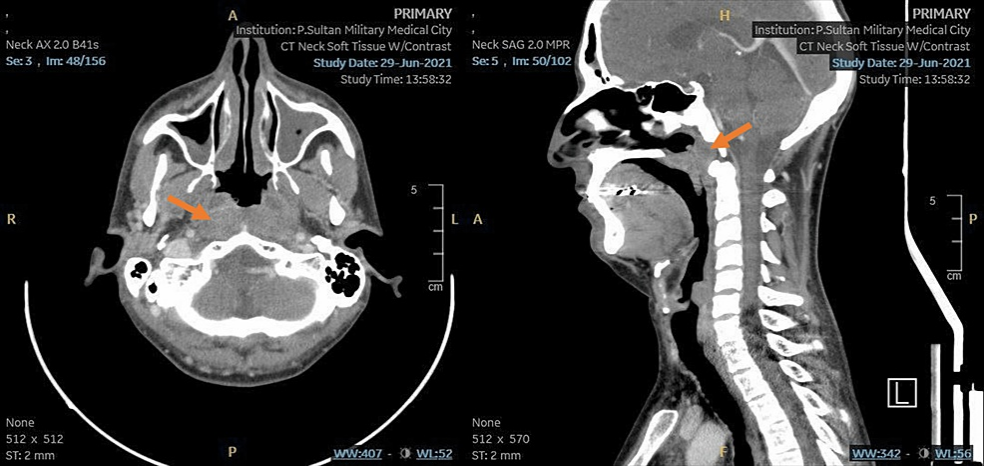
Contrast-enhanced CT of the head and neck, showing a relatively large ill-defined infiltrative heterogeneously enhancing soft tissue mass that is noted in the nasopharynx (orange arrow). It obliterates the fossae of Rosenmuller and pharyngeal openings of both Eustachian tubes with a marked widening of the posterosuperior nasopharyngeal wall. CT, computed tomography

The patient underwent a contrast-enhanced CT of the head and neck, which showed a relatively large ill-defined infiltrative heterogeneously enhancing soft tissue mass that is noted in the nasopharynx (Figure 1). It obliterates the fossae of Rosenmuller and pharyngeal openings of both Eustachian tubes with a marked widening of the posterosuperior nasopharyngeal walls. The mass extends anteriorly through the choanae into the posterior nasal cavity, mainly along the right side. Posteriorly, it causes skull base bony erosive changes mainly along the anterior clivus, which is markedly eroded. Laterally, the mass extends to the right pterygomaxillary fissure and right pterygopalatine fossa, which is markedly widened by the soft tissue density. The right compartment of the sphenoid sinus is opacified with a hyperdense material associated with bony erosions at the floor, which is likely secondary to retained secretions rather than a tumoral extension. The possibility of secondary sphenoidal fungal infection cannot be ruled out. No intracranial or intraorbital extensions. Associated large bilateral cervical lymphadenopathy is seen mainly implicating left IIA, bilateral IIB, III, and IV levels, more on the left side. The enlarged lymph nodes show heterogeneous enhancement, and some of them show central necrosis. The largest enlarged node on the left side measures about 3.1 cm × 2.1 cm in maximum dimension.

An endoscopic-guided biopsy was taken from the mass after the CT report for histopathological confirmation. The biopsy revealed an undifferentiated, non-keratinizing NPC that is positive for EBV.

The patient had a CT chest-abdomen-pelvis as a staging workup, which revealed no intrathoracic nor intraabdominal metastasis.

The case was reviewed by the tumor board, the patient was diagnosed with a stage III (T3N2M0; American Joint Committee on Cancer [AJCC], eighth edition) NPC and a decision was made for induction chemotherapy followed by radiotherapy. The management plan was to start induction chemotherapy (Taxotere/Carboplatin) for three cycles, one cycle every three weeks, followed by radiotherapy. The patient and his family attended the oncology clinic, got the management plan explained by the oncology team, and agreed to start. In total, the patient received three cycles of chemotherapy (Taxotere/Carboplatin) and 33 cycles of radiotherapy. The patient has been following up for two years with no residual headache nor evidence of recurrence observed.

## Discussion

NPC commonly presents with a variety of symptoms that may be nonspecific. The most common presenting symptom is neck mass. Other symptoms include unilateral ear block or reduced hearing, recurrent epistaxis, infrequently, nonspecific headaches, dysphagia, tinnitus, facial numbness, diplopia, nasal obstruction, and weight loss. The nonspecific nature of the disease can sometimes lead to a delay in early diagnosis when the condition is in its early stages. This delayed diagnosis may negatively affect the prognosis of the patient with NPC. Because of the vague symptoms that patients with NPC commonly present with, it is indicated to perform nasopharyngoscopy in any adult patient who presents with unilateral ear symptoms, recurrent epistaxis, neck mass or headaches, and facial pain [17]. Lee and Ho reported 14 NPC patients who presented solely with a headache. The average duration of headaches before NPC diagnosis was 7.9 months. The location of the headache was commonly described as temporal or parietal. Although most patients in the study had T3 or T4 tumors, the five-year overall survival did not differ from other patients with NPC in their study [18]. Wu et al. have found that those who present only with headaches had the highest misdiagnosis rate of 86.4% among patients with nasopharyngeal cancer [19]. Velayutham et al. reported a case of a 45-year-old female that presented with a right hemicranial headache for two years and was diagnosed later to have an NPC [20].

It is strongly recommended to evaluate the nasopharynx in adult patients that present with unilateral otitis media with effusion, unilateral otolaryngology symptoms, or neck mass. There are, however, no clear guidelines on when to do a nasopharyngoscopy or refer the patient to someone that can perform a nasopharyngoscopy if the patient presents with a headache that is not in the distribution of the paranasal sinus (facial headache). If there is a lesson to be taken from this paper and similar papers, it is never to underestimate the headaches. Further data is needed to identify patients who present with a headache that should undergo an urgent nasopharyngoscopy.

## Conclusions

NPC is rare cancer that is difficult to diagnose due to its different irrelative presenting symptoms. Most of the time, it presents multiple symptoms, which can mislead physicians and, therefore, delay the appropriate treatment, which affects the survival rate. Headache alone can be the sole presenting symptom in some cases, such as the case that we presented in this paper, and it should not be taken lightly, especially if it is progressive and not responding to pain medications or if it is associated with red flag symptoms. Further data is needed to identify patients that present with a headache that should undergo an urgent nasopharyngoscopy to diagnose or rule out NPC.
